# COGNITIVE RESERVE AND RISK OF MOBILITY DISABILITY IN OLDER ADULTS

**DOI:** 10.1093/geroni/igac059.787

**Published:** 2022-12-20

**Authors:** Roee Holtzer, Xiaonan Zhu, Andrea Rosso, Caterina Rosano

**Affiliations:** Yeshiva University and Albert Einstein College of Medicine, New York, New York, United States; University of Pittsburgh, Pittsburgh, Pennsylvania, United States; University of Pittsburgh, Pittsburgh, Pennsylvania, United States; University of Pittsburgh, Pittsburgh, Pennsylvania, United States

## Abstract

**Background:**

Cognitive Reserve (CR) protects against cognitive decline and dementia but its relation to mobility disability has not been established. To address this important gap in the literature, we conducted a longitudinal investigation to test the hypothesis that higher baseline CR was associated with a lower risk of developing mobility disability in older adults.

**Methods:**

Participants were dementia-free older adults who received a brain magnetic resonance imaging and had gait speed assessments during follow-up. Using the residuals approach, CR was derived from the modified-Mini-Mental Status Examination (3MS) total score by removing variance accounted for by measures of structural brain integrity, education and race. Mobility disability was defined using a validated cutoff score in gait speed of 0.8 m/s. Logistic regression models using General Estimating Equations (GEE) were utilized to examine longitudinal associations between baseline CR and the risk of developing of mobility disability across repeated assessments.

**Results:**

Of the participants (n=237; mean age=82ys; %female=56%) who were free of mobility disability at baseline, 103 developed mobility disability during follow-up (mean=3.1ys). Higher CR at baseline was associated with lower risk of developing incident mobility/disability [odds ratio (OR)=.819, 0.67 to 0.98, p=.038 (unadjusted); OR=.815, 0.67 to 0.99, p=.04 (adjusted for socio-demographic variables and depression); OR=.819, 0.68 to 0.88, p=.035 (adjusted for illness history); OR=.824, 0.68 to 0.99, p=.045 (adjusted for white matter hyperintensities); OR= .795, 0.65 to 0.95, p=.016 (adjusted falls history)].

**Conclusion:**

We provided first evidence that higher CR was associated with lower risk of developing mobilitydisability in older adults.

